# Cochlear Implantation in a Patient with a Novel *POU3F4* Mutation and Incomplete Partition Type-III Malformation

**DOI:** 10.1155/2020/8829587

**Published:** 2020-09-01

**Authors:** Xiuhua Chao, Yun Xiao, Fengguo Zhang, Jianfen Luo, Ruijie Wang, Wenwen Liu, Haibo Wang, Lei Xu

**Affiliations:** Department of Otolaryngology-Head and Neck Surgery, Shandong Provincial ENT Hospital, Cheeloo College of medicine, Shandong University, Jinan 250022, China

## Abstract

**Aims:**

This study is aimed at (1) analyzing the clinical manifestations and genetic features of a novel *POU3F4* mutation in a nonsyndromic X-linked recessive hearing loss family and (2) reporting the outcomes of cochlear implantation in a patient with this mutation.

**Methods:**

A patient who was diagnosed as the IP-III malformation underwent cochlear implantation in our hospital. The genetic analysis was conducted in his family, including the whole-exome sequencing combined with Sanger sequencing and bioinformatic analysis. Clinical features, preoperative auditory and speech performances, and postoperative outcomes of cochlear implant (CI) were assessed on the proband and his family.

**Results:**

A novel variant c.400_401insACTC (p.Q136LfsX58) in the *POU3F4* gene was detected in the family, which was cosegregated with the hearing loss. This variant was absent in 200 normal-hearing persons. The phylogenetic analysis and structure modeling of Pou3f4 protein further confirmed that the novel mutation was pathogenic. The proband underwent cochlear implantation on the right ear at four years old and gained greatly auditory and speech improvement. However, the benefits of the CI declined about three and a half years postoperation. Though the right ear had been reimplanted, the outcomes were still worse than before.

**Conclusion:**

A novel frame shift variant c.400_401insACTC (p.Q136LfsX58) in the *POU3F4* gene was identified in a Chinese family with X-linked inheritance hearing loss. A patient with this mutation and IP-III malformation could get good benefits from CI. However, the outcomes of the cochlear implantation might decline as the patient grows old.

## 1. Introduction

Congenital deafness affects approximately 1 in 1,000 newborns. It is estimated that 20% of congenital hearing loss was caused by inner ear malformations (IEM) [1]. The etiology of IEM remains unknown. In mammals' inner ear, the cochlear hair cells transform the mechanical vibration of sound waves into the acoustic electrical information [2–5], while spiral ganglion neurons (SGNs) further deliver the acoustic electrical information all the way to the auditory cortex to establish the hearing ability. Many previous studies have reported that hair cell malformation caused by genetic factors, ototoxic drugs, aging, noise, and inflammation leads to IEM and hearing loss [6–9]. Incompletely partition type III (IP-III) is a relatively rare IEM that accounts for approximately 2% of all cochlear malformations [10]. The diagnosis of IP-III cochlear malformation is mainly based on its unique imaging features. The characters of temporal bone high-resolution CT (HRCT) in patients with IP-III malformation include the enlargement of the lateral end of the internal auditory canal (IAC), absence of the cochlear modulus, partial hypoplasia of the cochlea, abnormally communication between the IAC and cochlea, and stapes fixation. This malformation was first described by Nance et al. in 1971 [11], then Sennaroglu and Saatci classified it as a type of cochlear abnormality with incomplete partition and named it as IP-III [12].

A previous study found that IP-III malformation was a disease associated with X-linked hereditary deafness caused by *POU3F4* gene mutation [13]. *POU3F4* gene mutation is the most common cause related to X-linked nonsyndromic deafness, which accounts for about 50% of all X-linked deafness patients [14]. Human *POU3F4* gene locates on chromosome Xq21.1. This gene encodes the protein with bipartite DNA-binding domains, which belong to a superfamily of POU domain transcription factors [15]. The transcription factor *POU3F4* binds DNA using a specific DNA-binding domain, which is divided into two subdomains, a POU-specific (POUs) and a POU homeodomain (POU_HD_). POU superfamily genes play an important role in organ formation and cell differentiation. In the mice, *POU3F4* is expressed in the mesenchyme cells surrounding the developing inner ear epithelium with limited or no expression in the hair cells or SGNs [16, 17]. It is critical for the development of the spiral limbus, the scala tympani, and the temporal bone [18, 19]. Studies in the *POU3F4* knockout mice suggested that *POU3F4* deficiency caused defects in otic fibrocytes and stria vascularis, both of which were essential for the sound transduction [20].

In the clinic, patients with *POU3F4* mutation present with IP-III malformation, mixed or severe to profound sensorineural hearing loss (SNHL). For patients with severe to profound SNHL, cochlear implantation is still a unique effective treatment. It has been reported that the outcomes of cochlear implant (CI) in patients with IP-III malformation were worse than those who have normal cochlea and varied greatly among individuals [16, 21, 22]. To date, it is still hard to predict the cochlear implantation outcomes in patients with severe cochlea malformations preoperatively. Furthermore, because of the absence of the cochlear modulus and the abnormally communication between the IAC and cochlea, the electrode array was easily inserted into the IAC and severe cerebrospinal fluid (CSF) gusher often occurred during the surgery [21]. Thus, it is still a challenge to do cochlear implantation in this kind of patient.

In this paper, we reported the cochlear implantation in a patient with IP-III malformation. The clinical characteristics and genetic analysis in his family were also displayed. This is the first study to identify a c.400_401insACTC(p.Q136LfsX58) in the *POU3F4* gene associated with X-linked hereditary deafness in Asians. By molecular testing, we provided definitive diagnosis and genetic counseling for this family and further enriched the pathogenic mutation spectrum of the *POU3F4* gene. Our results also shed light on the potential use of IP-III malformation genotypes as meaningful biological markers of the outcome of CI.

## 2. Materials and Methods

### 2.1. Subjects

This study was approved by the Shandong University ethical committee (number 014). All participants involved in the project signed written informed consent. A Chinese family affected by X-linked inheritance hearing loss was recruited. The proband came to our hospital when he was four years old due to hearing loss and no speech. Besides, 200 persons with normal hearing were collected. All audiometric tests and physical examinations were evaluated at Shandong Provincial ENT Hospital.

### 2.2. Clinical Evaluations

The medical history of the proband was obtained, including health condition at birth, newborn hearing screening, onset and progress of hearing loss, otitis media and ototoxic drug using history, hearing aid using history, maternal health during pregnancy, and other relevant clinical manifestations to exclude any history of other diseases and environmental factors. He underwent a series of clinical tests including physical examinations, distortion product otoacoustic emission, tympanometry, pure tone audiometry, auditory brainstem response (ABR), and temporal bone MRI and CT scans.

### 2.3. Genetic Analyses

Genomic DNA of each member in the family and 200 normal-hearing controls were extracted from the peripheral blood using a DNA extraction kit (AXYGEN). The common mutations of *GJB2*, *SLC26A4*, and mtDNA 12S rRNA genes were excluded by the “SNPscan assay” (Genesky Biotechnologies Inc., Shanghai, China). Whole-exome sequencing (WES) was used to investigate genetic variations underlying the hearing loss in the family. Genomic DNA was fragmented to 180~280 bp, and the DNA library was created following established Illumina paired-end protocols. The Agilent SureSelect Human All ExonV6 Kit (Agilent Technologies, Santa Clara, CA, USA) was employed for exome capture according to the manufacturer's instructions. The Illumina NovaSeq 6000 platform (Illumina Inc., San Diego, CA, USA) was used for genomic DNA sequencing by Yinfeng Gene Technology Co., Ltd. (Beijing, China) to generate 150 bp paired-end reads with a minimum coverage of 10× for ~97% of the target sequence (mean coverage of 100×). The resulting fastq data were analysed by in-house quality control software to remove low-quality reads and were then aligned to the reference human genome (hs37d5) using the Burrows-Wheeler Aligner [23], and duplicate reads were marked using Sambamba tools [24]. Annotation was performed using ANNOVAR [25]. After whole-exome sequencing, we identified one candidate mutation. The PCR and Sanger sequencing were performed to determine whether the variant was cosegregated with hearing loss in the family. The following primers were synthesized: 5′-GCCTAATTTGGAAAGCGAGC-3′ and 5′-AAATCCGCGCTGCTCCCAGT-3′ (BGI Inc., China). The PCR and amplification were performed according to a previous protocol [26]. The sequence of *POU3F4* fragment was performed using the DNASTAR software.

### 2.4. Phylogenetic and Structural Analyses

Phylogenetic analysis of Pou3f4 was performed with multiple sequence alignment using BioEdit software. The sequences included NP_000298.3 (Homo sapiens), NP_032927.1 (Mus musculus), NP_058948.1 (Rattus norvegicus), NP_001181188.1 (Macaca mulatta), XP_003317585.1 (Pan troglodytes), XP_010820097.1 (Bos taurus). Three-dimensional (3D) modeling of the human wild-type and mutant POU3F4 protein was carried out using I-TASSER (http://zhanglab.ccmb.med.umich.edu/). The wild-type domain includes 361 amino acids (NP_000298.3), and the mutant domain includes 192 amino acids. Predicted wild-type and mutant protein structures were observed and analyzed using PyMOL visualization software.

### 2.5. Cochlear Implantation

A standard transmastoid facial recess approach was used for cochlear implantation. The 1 J electrode array (Advanced Bionics Corp., Sylmar, CA) was implanted in the proband on the right ear when he was four years old. The electrode array was fully inserted. Four years post the first implantation, the right ear had been reimplanted with a new 1 J electrode array due to the deterioration of the cochlear implant benefits. He underwent the third cochlear implantation on the left ear when he was nine and a half years old because his parents were not satisfied with the outcomes of the reimplantation. The left ear was also implanted with the 1 J electrode array. The electrode arrays were fully inserted in both ears.

During the operation, CSF gusher occurred upon opening the round window. After the insertion of the electrodes, the cochleostomy was blocked using prepared muscle tissue. No CSF leakage was observed after surgery. Intraoperative CT scan was utilized to ensure the electrode array at the correct position and did not insert into the IAC. No complications were observed postoperatively. He came back regularly for programming and evaluation. Aided hearing thresholds and speech perception were tested to evaluate the outcomes of cochlear implants. Besides, the auditory and speech ability were accessed using categories of auditory performance (CAP) and speech intelligibility rating (SIR).

## 3. Results

### 3.1. Clinical Features

**Figure 1:**
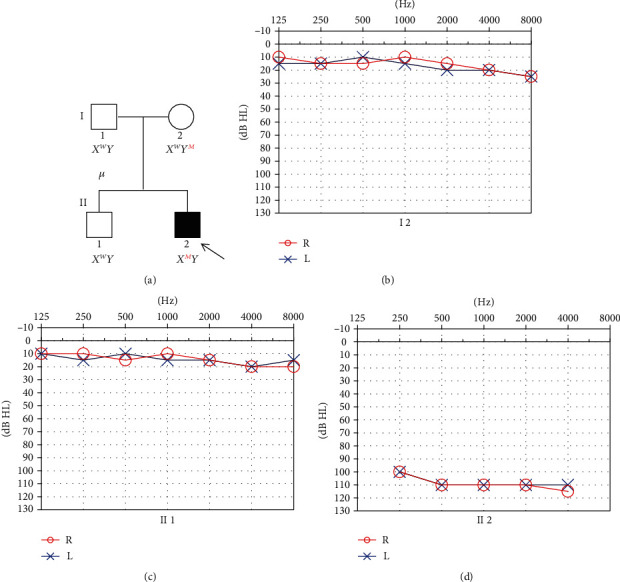
Clinical phenotype presentations of the pedigree. (a) Pedigree of a Chinese family displaying X-linked inheritance hearing loss (arrow indicates the proband; mutation c.400_401insACTC in *POU3F4* gene denoted as M); (b–d) Pure-tone audiograms of the family. Frequency in hertz (Hz) is plotted on the *x*-axis and the hearing level in decibels (dB HL) on the *y*-axis.

**Figure 2:**
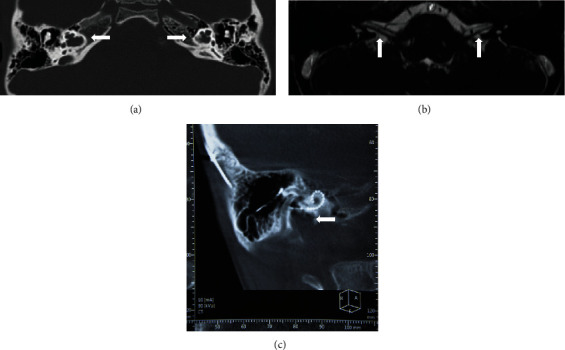
(a) Temporal bone CT images of the proband demonstrating dilation of the bottom of the internal auditory canal (IAC), bony plate deficition between the basal turn of the cochlea and the IAC, absence of the modiolus, but the interscalar septa of the cochlea are present (arrow); (b) Axial MRI through the IAC displaying the vestibulocochlear nerve and the facial nerve; (c) Three-dimensional reconstruction of the cochlea showed that the electrode array was in the correct position.

The proband (II-2) and three unaffected members in his consanguineous family were enrolled in this study (Figure 1(a)). The proband failed to pass the newborn hearing screening. He began to use hearing aids on both ears since one year old. The otoacoustic emissions were bilaterally absent. The ABR thresholds with clicks were 70 dB nHL in the left ear and 95 dB nHL in the right ear when he was one year old. No ABR response could be found on both ears at 100 dB nHL before the operation. Pure tone audiometry showed bilateral profound SNHL (Figure 1(d)). He has normal external ear and tympanic membrane. Acoustic immittance showed type A tympanograms in both ears. High-resolution temporal bone CT scan revealed the absence of the bony modiolus, hypoplasia of cochlea, enlarged internal acoustic canal, abnormal communication between the internal acoustic canal and the cochlear, and vestibular abnormalities (Figure 2(a)). MRI showed a normal internal auditory canal; the facial nerve and vestibule cochlear nerve could be seen clearly on both sides (Figure 2(b)). Pure tone audiometry of the proband's mother and brother were shown in Figures 1(b) and 1(c). All the proband's parents and brother have normal hearing, denying a family history of hearing loss.

### 3.2. Identification of the Novel Mutation c.400_401insACTC in *POU4F3*

**Figure 3:**
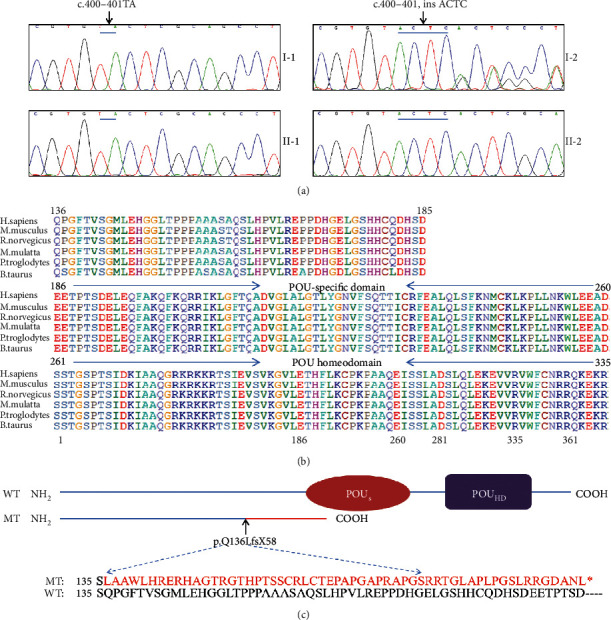
Partial sequence chromatograms, conservation analysis, and schematic illustration of Pou3f4. (a) DNA sequencing profile showing the c.400_401insACTC mutation in *POU3F4*. The sequence chromatograms were analyzed from PCR products of four family members. The arrow indicates the location of the nucleotide changes. (b) Protein alignment showing conservation of part residues of Pou3f4 across six species. (c) Schematic illustration of Pou3f4 protein labeled with the mutation identified in this study. The mutation introduces frame shift and a premature stop codon at amino acid 194, resulting in the nonfunctional protein without the POUs and POU_HD_ domains.

After the whole-exome sequencing in family members, an insertion mutation c.400_401insACTC (p.Q136LfsX58) in the *POU3F4* gene was detected in the proband suggesting a potential disease-causing factor in his family. In order to verify that the mutation was cosegregated with the hearing loss, the direct sequencing of the mutation site of the *POU3F4* gene in all family members was conducted. As shown in Figure 3(a), the identified novel mutation c.400_401insACTC in *POU4F3* mutation cosegregates with hearing loss in this family. The mother of the proband (I-2) with this heterozygous mutation presented normal hearing. The variant was not present in two hundred normal hearing controls. The mutation leads to a reading frame shift at amino acid position 136 and results in a premature termination in *POU3F4* (Figure 3(c)). As shown in Figure 3(b), the protein sequences were highly evolutionarily conserved and implicated to have a significant functional consequence. The dysfunction of the truncated protein without the DNA-binding domains of POUs and POU_HD_ might contribute to the observed phenotype in the affected member of the Chinese family.

**Figure 4:**
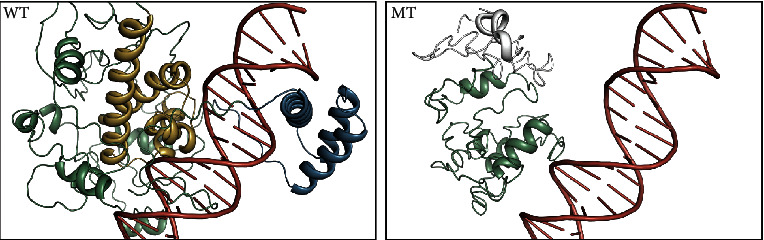
Structural simulation of Pou3f4. Compared with the wild-type (WT) protein, the structure of the mutant protein is incomplete, leading to lose the DNA-binding ability. The POUs (yellow) and POU_HD_ (blue) domains are shown in different colors.

A molecular model of *POU3F4* was constructed based on the crystal structure of paired box domain (PDB ID: 2XSD). The predicted structures of wild-type and mutant proteins were observed and analyzed using PyMOL. As shown in Figure 4, the protein Pou3f4 bound to the DNA elements with POUs and POU_HD_ domains. Compared with the wild-type structure of the Pou3f4, the truncated protein showed elimination of the specific DNA-binding domain, leading to lose the DNA-binding ability.

### 3.3. Outcomes of Cochlear Implantation

**Figure 5:**
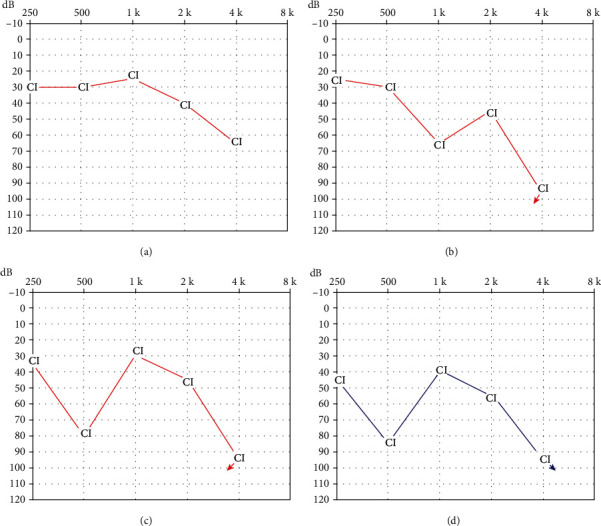
Audiometric thresholds with cochlear implants at different time points in different ears. (a) and (b) were hearing threshold on the right ear tested three and four years post the first implantation; (c) Hearing threshold on the right ear tested two years post the reimplantation; (d) Hearing threshold on the left ear tested half a year post the implantation. CI means hearing threshold with cochlear implant; arrow means no response.

The proband gained great improvement on the auditory and speech ability after the first implantation. The audiometric thresholds improved to approximately 30 dB HL across all speech frequencies except the 4 kHz (Figure 5(a)). The CAP scale improved from 1 to 6, and the SIR scale improved from 1 to 4 at three years post the first implantation. The speech recognition tested three years postoperatively showed monosyllable words score was 61%, and bisyllable words score was 55%. He studied in an ordinary school, and the main mode of communication was verbal language. Approximately three and a half years post the first implantation, the benefits of the cochlear implant began to decline. His parents noticed that he began to have slurred speech. The hearing threshold at the 1 kHz increased greatly, and there was no response at 4 kHz (Figure 5(b)). He began to use lip reading to assist communication. The temporal bone CT scan showed that the electrode array was at the correct position in the cochlea (Figure 2(c)). The impedance of the electrodes was stable and in normal range. The speech processor was in good condition. Though the cochlear implant had been programmed several times, the outcomes were still worse than before.

The hearing threshold with the cochlear implantation on the right ear at two years post the reimplantation was displayed in Figure 5(c). The patient got little benefit at the frequency of 500 Hz and still has no response at 4 kHz. The speech recognition tested two years postoperatively showed monosyllable words score was 20%, and the bisyllable words score was 13%. The hearing threshold with the cochlear implantation on the left ear six months postoperatively was displayed in Figure 5(d). Similar hearing threshold frequency shape was seen on the left ear as it is shown on the right ear. The speech recognition tested on the left ear showed monosyllable words score was 48%, and the bisyllable words score was 27%. Better speech perception was obtained with bilateral cochlear implants: monosyllable words score was 56%, and the bisyllable words score was 47%. Facial nerve stimulation by cochlear implantation was seen in the right ear.

## 4. Discussion

In this study, we identified a novel mutation c.400_401insACTC in the *POU3F4* gene causing SNHL loss in a X-linked recessive Chinese family combined WES and Sanger sequencing. The c.400_401insACTC mutation resulted in frame shift at amino acid position 136 located in upstream of the POU structure (p.Q136LfsX58). This frame shift caused a premature termination resulting in a protein lacking the entire POU homeodomain and specific homeodomain. Compared with the wild-type structure of the Pou3f4, the truncated protein caused by c.400_401insACTC mutation severely disrupts the DNA binding ability of *POU3F4*. Thus, the transcriptional activity of this truncated Pou3f4 protein has been completely abolished. Previous studies indicated that the hearing loss in patients with *POU3F4* mutation was caused by the functional deficit of the Pou3f4 protein [27, 28]. In this study, the proband showed specific IP-III malformation and progressive hearing loss. To date, more than 70 pathogenic variants of the POU3F4 gene have been reported in the Human Gene Mutation Database, including intragenic mutations, complete or partial deletions, duplications, insertions, and other chromosomal deletions. Most of the pathologic mutations are deletion or intragenic mutations, and only a few are inversion mutations. Moteki et al. also reported an insertion mutation (c.727_728insA) in *POU3F4* which also caused progressive hearing loss and IP-III malformation [29]. The severity of hearing loss among patients with different types of *POU3F4* mutations varied greatly. To date, no correlation was found between certain genotypes and initial auditory phenotype [28, 30].

The proband underwent sequentially cochlear implantation on both ears. The auditory and speech ability improved greatly after the first cochlear implantation. Three years postoperatively, the CAP score was 6, which meant that the patient can communicate with others without lip reading. Besides, the SIR score improved to 4 which meant that his pronunciation was clearly enough that could be understood by an acquaintance. However, the speech recognition scores with CI on both ears were lower than previously reported patients who have normal cochlea [31, 32]. It has been reported that the outcomes of cochlear implantation in patients with IP-III malformation were generally poorer than patients with normal cochlea [22, 27, 33]. Electrical stimuli delivered by the CI are first encoded by the SGNs, and subsequently transmitted to, and processed by, higher-level neural structures. Thus, the ability of the SGNs to faithfully encode and process electrical information is critical for CI outcomes. It has been demonstrated that the number and capability of the SNGs is an important factor for CI outcomes [34–36]. Animal studies showed that the radial bundle fasciculation and hair cell innervation of the SGNs were impaired after Pou3f4 is deleted from otic mesenchyme [34]. Presumably, the responsiveness of SGNs to electric stimuli was impaired in patients with *POU3F4* mutation. Furthermore, the benefits of CI varied greatly among individual patients with IP-III malformation. Stankovic et al. reported that CI had limited benefits on the auditory and speech perception in patients with IP-III malformation [22]. In contrast, Kim et al. reported that patients with IP-III malformation acquired good speech after cochlear implantation [33]. Factors accounting for these variations are still not well understood. Choi et al. reported that the *POU3F4* genotype might be an important factor for the outcomes of cochlear implantation [27]. In that study, patients with the *POU3F4* truncation or deletion mutations had poorer speech performance than patients with other types of mutations [27]. In our study, the insertion mutation c.400_401insACTC in the *POU3F4* gene resulted in the loss of the conserved domains of Pou3f4 protein. However, the patient presented good outcomes with a cochlear implant. Many factors might contribute to the contradictory results, such as the type of electrode array, implantation age, and follow-up time were different in these two studies. Besides, only few patients were included in these two studies, and more data were needed to investigate the relationship of the *POU3F4* genotype and the outcomes of cochlear implantation.

In this study, we firstly reported that the outcomes of the cochlear implantation in patients with IP-III malformation would decline. Since the position of the electrode array, the device and processor were in good condition, we speculated that deterioration of the benefits was caused by the changes of the function of the SGNs in the cochlea. It has been reported that the SGNs density begins to decline by the end of the first postnatal week in *POU3F4* knock out mouse [37]. To date, there were no direct evidences that showed that the function of the SGNs would decline in patients with *POU3F4* mutation. However, it has been reported that patients with *POU3F4* mutation often presented with progressive hearing loss [38], which might indicate that the function of the SGNs would decline with age.

After the deterioration of the CI benefits, the hearing thresholds were only increased in some specific frequencies. This indicated that the number and responsiveness of the SGNs might vary along the cochlea. Because of the absence of the modulus in patient with IP-III malformation, the distribution and function of residual SGNs in the cochlea are still unclear. In this study, the patient had no response on 4 kHz on both ears, which indicated that the function of the SGNs around the basal electrodes might be worse than others. This was consistent with previously reported results. Stankovic et al. has reported the outcomes of cochlear implantation in four patients with *POU3F4* mutation; none of them could perceive sound from the basal electrodes [22]. Before the reimplantation, the proband showed bad response on 1 kHz and 4 kHz. However, he showed high threshold on 500 Hz and 4 kHz after the reimplantation. This might be due to the insertion depths of the electrode array in those two operations that were different. Thus, the stimulating frequencies to the SGNs had been reshaped.

## 5. Study Limitations

This study has several potential limitations. One potential limitation is that the neurophysiological mechanisms underlying the deterioration of the CI outcomes had not been assessed. Currently, the electrically evoked compound action potential (eCAP), a near-field neural response generated by the SGNs, has been widely used to evaluate the functional status of the SGNs [36]. The slope of eCAP input/output (I/O) function and neural refractoriness are associated with the density and function of the surviving neural population [32]. Besides, the maximum eCAP amplitude and eCAP threshold are also determined by the number of CN fibers activated by electrical stimuli [39]. In this study, the proband only did the introoperation neural response imaging (NRI). However, it has been reported that the NRI thresholds post the first half-year of implantation cannot faithfully reflect the response of the SGNs. In addition, none eCAP input/output (I/O) function data were collected before the reimplantation. Therefore, we failed to compare the function of SGNs before and post the deterioration of the CI benefits. More comprehensive eCAP responses would be assessed to follow-up on the functional changes of the SGNs in the future.

## 6. Conclusion

In this study, a novel mutation c.400_401insACTC (p.Q136LfsX58) in the *POU3F4* gene was identified in a Chinese family with X-linked recessive hearing loss. The molecular genetic research showed the association between X-linked hearing loss and mutations in *POU3F4*, providing the definitive diagnosis and genetic counseling for this family and further enriched pathogenic mutation spectrum of the *POU3F4* gene. The patient with this variant showed specific IP-III malformation and progressive hearing loss. Patients with this mutation could get benefits form cochlear implantation. However, the outcomes of the cochlear implantation might decline as the patient grows old. The mechanism of the effect of the *POU3F4* genotype on the distribution and function of SGNs in IP-III malformation deserves further investigation.

## Data Availability

All the data in this study were collected in Shandong Provincial ENT Hospital. The processed data are available within the article.
